# PLGA Nanoparticles Containing Natural Flavanones for Ocular Inflammation

**DOI:** 10.3390/pharmaceutics15122752

**Published:** 2023-12-11

**Authors:** Paola Bustos-Salgado, Valeri Domínguez-Villegas, Berenice Andrade-Carrera, Mireia Mallandrich, Ana Calpena, Oscar Domènech, Sergio Martínez-Ruiz, Josefa Badía, Laura Baldomà, Inmaculada Gómez de Aranda, Juan Blasi, María Luisa Garduño-Ramírez

**Affiliations:** 1 Departament de Farmàcia i Tecnologia Farmacèutica, i Fisicoquímica, Facultat de Farmàcia i Ciències de l’Alimentació, Universitat de Barcelona (UB), Av. Joan XXIII 29-31, 08028 Barcelona, Spain; paola_bustos_salgado@ub.edu (P.B.-S.); bereniceac@uaem.mx (B.A.-C.); anacalpena@ub.edu (A.C.); odomenech@ub.edu (O.D.); 2 Institut de Nanociència i Nanotecnologia (IN2UB), Universitat de Barcelona (UB), 08028 Barcelona, Spain; 3 Facultad de Ciencias Químicas e Ingeniería, Universidad Autónoma del Estado de Morelos, Av. Universidad 1001, Cuernavaca 62209, Morelos, Mexico; valeri.dominguez@uaem.mx; 4 Facultad de Nutrición, Universidad Autónoma del Estado de Morelos, Calle Iztaccihuatl S/N, Col. Los Volcanes, Cuernavaca 62350, Morelos, Mexico; 5 Department de Bioquímica i Fisiologia, Facultat de Farmàcia i Ciències de l’Alimentació, Universitat de Barcelona, 08028 Barcelona, Spain; sergio_martinez_ruiz@ub.edu (S.M.-R.); josefabadia@ub.edu (J.B.); lbaldoma@ub.edu (L.B.); 6 Institute of Biomedicine of the University of Barcelona (IBUB), 08028 Barcelona, Spain; 7 Research Institute Sant Joan De Déu (IR-SJD), 08950 Barcelona, Spain; 8 Departament de Patologia i Terapèutica Experimental, Facultat de Medicina i Ciències de la Salut, Bellvitge Campus, Universitat de Barcelona, 08907 Hospitalet de Llobregat, Spain; igomezdearanda@ub.edu (I.G.d.A.); blasi@ub.edu (J.B.); 9 Centro de Investigaciones Químicas, Instituto de Investigación en Ciencias Básicas y Aplicadas, Universidad Autónoma del Estado de Morelos, Av. Universidad 1001, Cuernavaca 62209, Morelos, Mexico; lgarduno@uaem.mx

**Keywords:** *Eysenhardtia platycarpa*, natural flavanones, PLGA nanoparticles, HPLC method validation, in vitro release, ex vivo skin permeation

## Abstract

Flavanones are natural compounds that display anti-inflammatory activity. The aim of this work was to prepare PLGA nanoparticles (NPs) containing natural flavanones I ((2*S*)-5,7-dihydroxy-6-methyl-8-(3-methyl-2-buten-1-il)-2-phenyl-2,3-dihydro-4*H*-1-Benzopyran-4-one) and II (2*S*)-5,7-dihydroxy-2-(4′-methoxyphenyl)-6-methyl-8-(3-methyl-2-buten-1-yl)-2,3-dihydro-4*H*-1-Benzopyran-4-one) (NP I and NP II, respectively) so as to evaluate their potential for topical anti-inflammatory ocular therapy. An in silico study was carried out using the Molinspiration^®^ and PASS Online web platforms before evaluating the in vitro release study and the ex vivo porcine cornea and sclera permeation. The HPLC analytical method was also established and validated. Finally, the in vitro anti-inflammatory efficacy of NPs was studied in the HCE-2 model. The flavanones I and II could be released following a kinetic hyperbolic model. Neither of the two NPs was able to permeate through the tissues. NP I and NP II were found to be respectful of any changes in the tissues’ morphology, as evidenced by histological studies. In HCE-2 cells, NP I and NP II were not cytotoxic at concentrations up to 25 µM. NP I showed higher anti-inflammatory activity than NP II, being able to significantly reduce IL-8 production in LPS-treated HCE-2 cells. In summary, ocular treatment with NP I and NP II could be used as a promising therapy for the inhibition of ocular inflammation.

## 1. Introduction

Ocular inflammation is one of the most common disorders in ophthalmology and is involved in many pathological conditions and diseases that affect the anterior or posterior segment of the eye [1,2,3]. The eyes are vulnerable as they are highly exposed to ultraviolet light, environmental damage and oxygen from the atmosphere. This fact can cause inflammation and oxidative damage that accelerates structural changes in ocular tissues. In consequence, this facilitates the development and progression of eye pathologies that can cause vision loss in extreme cases [4]. The eyelids, conjunctiva, cornea and uvea can be affected when ocular inflammation occurs [1,5]. Current pharmacological treatment for the management of ocular inflammatory diseases includes topical administration of steroidal anti-inflammatory drugs (SAIDs) and non-steroidal anti-inflammatory drugs (NSAIDs) [6,7,8]. In any case, it is known that the prolonged use of this type of drugs can cause various side effects. All of the foregoing provides reasons why it is necessary to carry out research to find safer and more effective therapies.

**Figure 1:**
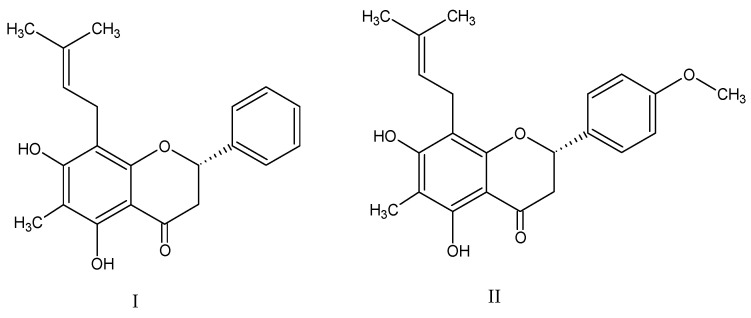
The molecular structure of flavanone I: (2*S*)-5,7-dihydroxy-6-methyl-8-(3-methyl-2-buten-1-il)-2-phenyl-2,3-dihydro-4*H*-1-Benzopyran-4-one) and flavanone II: (2*S*)-5,7-dihydroxy-2-(4′-methoxyphenyl)-6-methyl-8-(3-methyl-2-buten-1-yl)-2,3-dihydro-4*H*-1-Benzopyran-4-one.

Flavanones, polyphenolic compounds, have a wide variety of biological effects such as antioxidant, antiviral, antibacterial, anti-inflammatory, anti-allergic and even anticancer [9,10,11,12,13,14], and are shown to be a potential complementary ocular treatment. Natural flavanones I and II (Figure 1), obtained by methanolic extraction from *Eysenhardtia platycarpa* leaves, have displayed antioxidant properties evaluated by the DPPH (2,2-diphenyl-2-picrylhydrazyl hydrate) radical scavenging assay. In addition, they have exhibited anti-inflammatory properties when working with the TPA-induced acute inflammation model in mice ears and, as shown with the cytotoxic effects on brine shrimp, with the *Artemia salina* method [15]. The anti-inflammatory property of flavanones could be beneficial in the treatment of ocular diseases.

In any case, the treatment of eye disorders is limited in part by the poor aqueous solubility of natural molecules, which comes from their chemical structure. On the other hand, the anatomy and physiology of the eye represents an effective barrier for the absorption of molecules [6]. This is in addition to the fact that the substances administered to the eyes are eliminated via different mechanisms (lacrimation, tear dilution and tear turnover), which results in a low bioavailability of the administered molecule [16]. Less than 5% of the active substance administered into the eye is retained on the ocular surface. To maintain an adequate therapeutic level of the drug used, frequent administration of drops is therefore necessary. However, this frequent use can induce toxic side effects and cell damage on the ocular surface [17]. For this reason, it is important to find new vehicles that solve both the solubility problem and the retention problem that restricts their bioavailability. An ideal formulation should be administered in the form of drops. Thus the release of the drug overcomes the protection barriers of the eye and without causing tissue damage, blurred vision or irritability [6]. One of the most successful approaches to overcoming these drawbacks is the use of nanostructured ophthalmic drug delivery systems to overcome the physiological barriers of the eye and increase the bioavailability and subsequently the therapeutic action of ocular drugs [16,18]. Nanoparticles, one type of these nanostructured systems, have several advantages, among which is their low irritability as they are small in size and the sustained release of the drug, thus avoiding frequent administration [19]. Nanoparticles can adhere to the ocular mucosa and remain in the anterior corneal tissue for a long time (approximately 2 months) without rapid clearance [20,21]. Polylactic-co-glycolic acid (PLGA) is one of the most studied synthetic polymers to produce nanoparticles due to its biocompatibility and biodegradability [22].

All of these are reasons for which the present study was designed to develop two PLGA nanoparticles containing natural flavanones I and II (NP I and NP II) to prevent and relieve ocular inflammation with topical application. An analytical method for flavanones I and II quantification using HPLC-UV range was developed and validated. Molinspiration^®^ and PASS (prediction of activity spectra for substance) online web analyses were carried out to find out if the flavanones I and II possessed an anti-inflammatory drug-like character. The physicochemical characteristics of each PLGA nanoparticle were studied as well as their drug release profile. The capacity of the flavanones in permeating the pig cornea and sclera was assessed by ex vivo permeation tests. To this end, histological analysis, cytotoxicity and the quantification of cytokine IL-8 were evaluated to provide evidence of the innocuousness and effectiveness of NPs on ocular inflammatory conditions.

## 2. Materials and Methods

### 2.1. Chemicals and Reagents

Sigma Aldrich (Madrid, Spain) supplied all the chemicals and reagents used in the present study. Purified and filtered water was obtained from MilliQ^®^ Plus System lab supply (Millipore Corporation; Madrid, Spain). Dialysis cellulose membrane molecular weight cutoff (MWCO) 12 kDa was acquired from Iberlabo (Madrid, Spain).

### 2.2. Plant Extraction

The flavanones I and II were obtained by methanolic extraction from *E. platycarpa* leaves as described in the previous reports put forward by our investigation group [23]. The leaves were collected in Tetipac, Guerrero, Mexico (Registration Number: Ramiro Cruz 1325 Sciences Faculty Herbarium Facilities of the Autonomous University of the State of Morelos) and then dried at room temperature, pulverized and extracted with methanol (three consecutive macerations: 100 g of dried vegetal material per 1000 mL methanol, at room temperature). Reduced pressure was used to remove the solvent and then the flavanones I and II were isolated by column chromatography at a reduced pressure. Finally, they were purified by thin-layer chromatography (TLC). The flavanones I and II were obtained as yellow powders and characterized by comparison with the previous published melting point data (174–177 °C and 152–154 °C, respectively) and with the ^1^H-NMR results [24].

### 2.3. Chromatographic Operating Conditions

High-performance liquid chromatography (HPLC) was used to firstly validate the method and then to determine the concentrations of the flavanones both in vitro and ex vivo diffusional analysis. The chromatographic system consisted of a Waters 515 HPLC pump, a 717 Plus autosampler, an analytical column Atlantis^®^ C18 5 μm 250 mm × 4.6 mm, Waters and a dual λ absorbance UV-VIS 2487 detector (Waters, Milford, MA, USA). It was used in isocratic mode at room temperature with 10 μL of sample injection volume, and a mobile phase of water and acetonitrile (80:20) at flow rate of 1 mL/min. The detection wavelength was 290 nm. The peak area was used to quantify each flavanone.

### 2.4. Analytical Method Validation

#### 2.4.1. Standard Solutions for Calibration Curves

Standard stock solutions of each flavanone (I and II, 100 µg/mL) were obtained daily diluting the appropriate amount of flavanone into a solution of water: ethanol (7:3). For the calibration curves, the standard stock solutions were diluted to reach the concentration ranges of 6.25, 12.5, 25, 50 µg/mL.

#### 2.4.2. Linearity

The one-way analysis of variance (ANOVA) test was used to evaluate the analytical linearity method by comparing peak areas versus nominal concentrations of each standard solution. If it turns out that there are no significant differences, then the method is linear. A significant difference is considered when *p* ˂ 0.05.

#### 2.4.3. Limit of Detection and Limit of Quantification

The limit of detection (LOD) refers to the lowest concentration that can provide a reliably distinguished LOD at a background level. Another question is that the LOQ is defined as the lowest concentration that can be precisely and accurately measured for an analyte. The limit of detection (LOD) and the limit of quantitation (LOQ) for each flavanone (I and II) were calculated using Equations (1) and (2) taking into account the standard deviation of the response and the slope of the calibration curve from six replicate analysis:

(1) $$\mathrm{LOD}=3.3\frac{SD_{Sa}}{S_{b}}$$

(2) $$\mathrm{LOQ}=10\frac{SD_{Sa}}{S_{b}}$$

where *SD_Sa_* is the standard deviation of the intercept and *S_b_* is the slope.

#### 2.4.4. Instrumental Repeatability

Standard stock solutions of flavanone I and II (100 µg/mL) were used to evaluate the instrumental repeatability by analyzing the sample seven times consecutively.

#### 2.4.5. Accuracy and Precision

The inter-day test was performed with samples of each flavanone I and II in three concentrations, 6.25, 25, and 100 μg/mL, once a day for six consecutive days. The accuracy and the precision of the analytical method were expressed as a relative error (RE%) and as the relative standard deviation (RSD%), respectively. Values of RE% and RSD% within ±15 were considered as an accurate and precise method. 

### 2.5. In Silico Analysis

The free web platforms Molinspiration^®^ [25] (http://www.molinspiration.com, accessed on 2 February 2023) and PASS Online [26] (Prediction of Activity Spectra for substance, http://way2drug.com/PassOnline, accessed on 2 February 2023) were used to predict the physicochemical properties and the probability that flavanones I and II would exert an anti-inflammatory effect (*Pa*).

### 2.6. Preparation of Flavanone Nanoparticles (NP I and NP II)

The flavanones I and II were loaded separately in a biopolymeric nanoparticle using the solvent displacement technique as described previously [27]. An organic solution containing poly-lactic-co-glycolic acid (PLGA 50:50, 0.09 g) and flavanone I or II (0.01 g) in 25 mL of acetone was poured under moderate stirring into 50 mL of an aqueous solution of poloxamer 188 (P188, 0.1 g), adjusted to pH 3.5 ± 0.05. The corresponding mixtures were evaporated to 10 mL under reduced pressure (Büchi, Labortechnik AG, Flawil, Switzerland) to yield the corresponding NP I and NP II formulations. Non-flavanone nanoparticle (nNP) was prepared following the same procedure described before but without adding any flavanone. The physicochemical characterization of NP I, NP II and nNP within the parameters of mean particle size (Z), polydispersity index (PDI), zeta potential (ZP) and encapsulation efficiency (EE) was previously described [23]. The values of each parameter were: Z, 173.26 ± 3.43 nm, 156.16 ± 2.28 nm and 202 ± 1.80 nm; PI values 0.03 ± 0.01, 0.05 ± 0.02 and 0.08 ± 0.01; ZP values of −26.65 ± 0.6 mV, −31.60 ± 0.2 mV and −25.70 ± 1.3 mV; EE values of 90 ± 4.30, 78 ± 3.02% for NP I, NP II and nNP, respectively.

### 2.7. Morphological Studies of Nanoparticles (SEM)

Scanning electron microscopy (SEM) was used to determine the shape and surface morphology of flavanones NPs after their preparation. One drop of each sample was deposited on a glass coverslip. After drying overnight, they were mounted on SEM holders and covered with a thin film of carbon to improve their conductivity. SEM studies were performed using a JSM-7001F (JEOL Inc., Peabody, MA, USA) on the TEM-SEM Electron Microscopy Unit from the Scientific and Technological Centers (CCiTUB), University of Barcelona. The software Image J 1.54d was used to measure the particle size from the SEM images [28].

### 2.8. Stability Study of Nanoparticles

The stabilities of nNP, NP I and NP II were studied by observing if any changes occurred in the NP size over time. Samples of NPs were stored at 4 ± 0.1 °C in a refrigerator for 8 weeks to determine the stability of the formulation. The Z, PDI and ZP of NPs were measured (*n* = 6) each week at 4 °C to evaluate the NP stability using a Zetasizer Nano ZS (Malvern Instruments, Malvern, UK). Samples were then directly placed into the module and the data were collected. Statistical analysis was performed using one-way analysis of variance (ANOVA) and the Tukey’s multiple-comparison post hoc test with an accepted level of significance of *p* < 0.05 value was carried out.

### 2.9. Osmolality

The osmolality of NP I and NP II was measured by means of Advanced Osmometer Model 3320 (Advance Instruments Inc., Norwood, MA, USA). The NP samples were placed directly into the Osmometer and the data were collected.

### 2.10. Transparency

The corneal transparency changes after NP I and NP II treatment were evaluated using an adapted methodology [29]. Corneas of pig were exposed to 500 µL of the following compound: 0.1 N NaOH as a positive control, 0.9% NaCl (physiological saline solution) as a negative control and NP I and NP II as the experimental formulations. Every 15 min, 500 µL of 0.9% NaCl were added until a maxim of 2 h to simulate a tear clearance. Positive and negative controls, the same for both formulations, were assayed in triplicate. The transmittance of the corneas was evaluated from 400 to 800 nm.

### 2.11. Ocular Irritation Study: Hen’s Egg Test-Chorioallantoic Membrane (HET-CAM)

The probable ocular irritation of the NP formulation was investigated by the hen’s egg test-chorioallantoic membrane (HET-CAM) method as described by Garrós et al. [30]. Fertilized hen’s eggs (from the G.A.L.L.S.A. farm, Tarragona, Spain) were used with a weight from 50 to 60 g. The previously incubated fertilized eggs were positioned in a climatic chamber at 37.0 ± 0.5 °C and 58% relative humidity in a horizontal position and rotated several times throughout the assay. Each egg was opened on the 10th day of incubation by cutting the shell towards the side where the air chamber was located. Subsequently, the inner membrane was removed, exposing the chorioallantoic membrane (CAM), and 150 µL of the tested compounds were deposited in each egg over the CAM: 0.1 N NaOH as the positive control, 0.9% NaCl (physiological serum) as the negative control, nNP formulation (without any flavanone), and NP I and NP II experimental formulations containing flavanone I and II, respectively. After, the behavior of the blood vessels was observed for 5 min, determining individually the time of the appearance of hemorrhage, lysis and coagulation. A different egg was used for each of the substances, carrying out each of the tests independently and in triplicate. Based on the measured times, the Irritation Index (IS, irritation score) was calculated using the following equation (Equation (3)):

(3) $$\mathrm{IS}=\left[\frac{\left(301-\mathrm{hemorrhage}\;\mathrm{time}\right)}{300}\times 5\right]+\left[\frac{\left(301-\mathrm{lysis}\;\mathrm{time}\right)}{300}\times 7\right]+\left[\frac{\left(301-\mathrm{coagulation}\;\mathrm{time}\right)}{300}\times 9\right]$$

For IS scores between 0 and 0.99 the substance is considered non-irritating, between 1.0 and 4.99 as slightly irritating, between 5.0 and 9.99 moderately irritating, and 10.0 to 21.0 as severely irritating.

### 2.12. Biological Tissues

The porcine corneas and sclera used in this study were obtained following the guidelines of the Ethics Committee for Animal Experimentation of the University of Barcelona (Spain) with the ethical approval code 515/18. The ocular tissues were removed from swine (weight 30–40 kg) in the practical laboratory and kept frozen at −78 ± 2 °C until use. The cryopreservation was carried out according to the internal protocols previously developed and approved by our research group [31].

### 2.13. In Vitro Analysis: Release

The diffusional Franz type was used to study the flavanone profile release from NPs. The system consisted of filling the receptor compartment with water: ethanol (7:3) solution at 32 ± 1 °C under continuous stirring. In the donator compartment, 300 µL of the corresponding flavanone NPs (NP I and NP II) were added. The dialysis membrane (12 kDa, Dialysis Tubing Visking; Medicell International Ltd., London, UK), hydrated 24 h before the release assay, separated the compartments with a diffusional area of 0.64 cm^2^. “Sink conditions” were maintained throughout the experiment. Samples of 300 µL were taken at different times over 45 h replenishing the same extracted volume every time. The flavanone concentrations were determined by HPLC (see Section 2.3), expressing the data as mean ± SD (*n* = 5). Data were evaluated following the kinetic model hyperbola using Equation (4):

(4) $$Q_{t}\;\left(\mu g\right)=\frac{\left(B_{max}\right)\left(t\right)}{\left(K_{d}+t\right)}$$

where *Q_t_* is the drug amount released at time *t* (µg); *K_d_* is the time needed to reach 50% of the drug released (h); and *B_max_* is the maximum amount susceptible to release (µg).

The Student’s *t*-test analysis was used to evaluate significant differences between the release profiles of both formulations. Data were considered statistically significant at *p* < 0.05.

### 2.14. Ex Vivo Analysis: Ocular Permeation

Franz-type diffusional cells were used to study the permeation flavanone profile through cornea and sclera of pigs (*n* = 5) in similar conditions as the in vitro analysis previously described (Section 2.13). In this case, the membrane consisted of cornea and sclera from pigs. Pig eyes were used as they are structurally the most human-like eyes [32]. The receptor compartment was filled with 3.7 mL of ethanol: water (70:30), and 300 µL of each NP formulation (NP I and NP II independently) were placed in the donor compartment covering them with Parafilm^®^ (Merck, Mollet del Vallès, Spain) to avoid evaporation. Samples were withdrawn at different points over 6 h and the amounts of each flavanone were quantified by means of a HPLC method, described in Section 2.3, to calculate the amounts of each flavanone permeated through the ocular tissues surface *Q_p_* (μg). Additionally, the area that was in contact with the NPs was cut, weighed, and pricked with a needle. Subsequently, 0.4 mL of the ethanol–water solution was added, and the mix was shaken in a sonic bath for 30 min. The resulting solution was quantified by the HPLC method (Section 2.3) and this gave the amount of flavanone retained in the cornea and sclera membrane (*Q_r_*, μg/cm^2^). At the end, Prism^®^, V. 5 software (GraphPad Software Inc., San Diego, CA, USA) was used to analyze the experimental data and to evaluate the significant differences between the permeation of the two different flavanones via analysis using Student’s *t*-test (data were considered statistically significant at *p* < 0.05).

### 2.15. Histology Evaluation

Corneas and sclera of pigs were collected separately in vials and then 500 µL of the substance to be tested were added: 0.1 N NaOH as a positive control, 0.9% NaCl (physiological saline solution) as a negative control and NP I and NP II were added as experimental formulations [29]. Every 15 min, 500 µL of 0.9% NaCl were added up to a maximum of 2 h, to simulate tear clearance. The experiment was carried out in triplicate. After that, the corneas and sclera were rinsed with PBS pH 7.4 and set for 24 h in 4% buffered formaldehyde. After fixation, samples were dehydrated, embedded in paraffin wax and sectioned on a microtome to obtain 5 µm transversal slices. Finally, the tissue sections were stained with hematoxylin and eosin on blind-coded samples before being analyzed and photographed using a Leica DMD108 microscope (Leica, Wetzlar, Germany).

### 2.16. Cell Viability Assays

Cell viability was assessed using the MTT (3-(4,5-Dimethylthiazol-2-yl)-2,5-diphenyl tetrazolium bromide) assay based on the mitochondrial reduction of tetrazolium to formazan. Formazan formation is considered directly proportional to the number of viable cells. This was followed by 100 µL of a human corneal epithelial cells (HCE-2) cell suspension of 1 × 10^5^ cells/mL being seeded in a 96-well plate and incubated for 24 h at 37 °C in the appropriate medium, after which it was treated. The cells were then tested with the different NPs (NP I, NP II, nNP) at concentrations ranging between 10 and 100 µg/mL and incubated for up to 48 h. The viable proliferating cells were measured using the MTT assay, as described previously [33]. Explaining this briefly, the cells were treated with 0.25% MTT (Sigma-Aldrich, Madrid, Spain) in PBS and allowed to react for 2 h at 37 °C. The medium was then removed and 1 mL of solubilization reagent (99% dimethyl sulfoxide) was added (Applichem, Ecogen, Barcelona, Spain). Cell viability was measured at 570 nm in a Modulus™ Microplate Photometer (Turner BioSystems, Sunnyvale, CA, USA). The results were expressed as the percentage of cell survival relative to the control (untreated cells).

### 2.17. In Vitro Evaluation of the NP Anti-Inflammatory Effects in LPS-Treated HCE-2 Cells

To evaluate the anti-inflammatory activity of the NP I and NP II, the LPS (lipopolysaccharide) inflammation model was used. To this end, HCE-2 cells were seeded at a density of 1 × 10^5^ cells/mL in 12-well plates and cultured until 90% confluence. Then, the cells were incubated with the NPs (nNP, NP I, NP II; 25 µg/mL) for 24 h, before the addition of LPS (10 µg/mL). After 24 h treatment, the culture supernatants were collected, centrifuged at 16,000× *g* for 20 min at 4 °C, and stored at −80 °C until use. Cells which had not undergone LPS treatment were processed as a negative control (CTRL). Secreted levels of the pro-inflammatory cytokine IL-8 were quantified by ELISA (enzyme-linked immunosorbent assay) (BD Biosciences, San Diego, CA, USA), in accordance with the manufacturer’s instructions. The results were expressed as pg/mL.

The data were collected from at least three independent biological experiments in triplicate. GraphPad Prism 5.0 software (GraphPad Software, Inc., La Jolla, CA, USA) was used to perform statistical analysis and generate graphs. The data are presented as the mean ± standard deviation (SD). Differences between the groups were assessed using a one-way analysis of variance (ANOVA), followed by Tukey’s post-test. Significant differences were established at a *p*-value < 0.05.

## 3. Results

### 3.1. Analytical Validation

Linearity can be expressed as the ability of an analytical method to obtain results that are directly proportional to the amount of the analyzed analyte in the sample [34]. The linearity of the HPLC method in the present study was determined by six calibration curves for the concentration range of 6.25–100 µg/mL for each flavanone. The results, as presented in Table 1, showed that the method is linear given that the coefficients of determination (r^2^) were greater than the 0.99 value for both flavanone I and II. In addition, no statistical differences were obtained (*p*-value > 0.05) after the analysis of variance (ANOVA) test of the corresponding calibration curves of flavanones I and II with *p*-values of 0.47 and 0.93, respectively. The flavanone II exhibited higher sensitivity than flavanone I with lower detection limits (LOD) and quantification limits (LOQ) (Table 1). The accuracy and precision calculated at three levels of concentration (maximum, medium and minimum) met the agreed criteria with relative error (RE) and relative standard deviation (RSD) values approximate 15% (Table 1). The instrument repeatability assessed showed an RSD % not greater than 8% for both flavanones.

**Figure 2:**
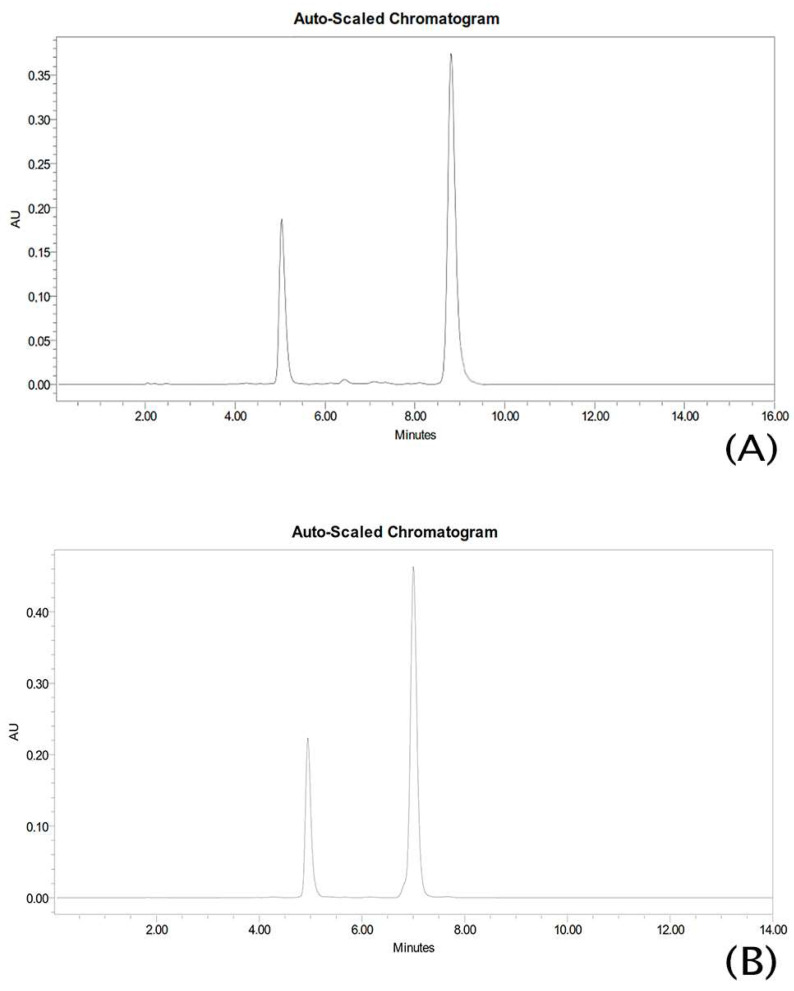
HPLC chromatograms (**A**) flavanone I and (**B**) flavanone II correspond to flavanone standard solution.

The chromatograms in Figure 2 show no interference from any other peak corresponding to each flavanone.

### 3.2. In Silico Analysis

The results of the theoretical measurements before in vitro assays with flavanones using the web platforms Molinspiration^®^ and PASS Online are shown in Table 2. The probability of flavanones I and II being active (*Pa*) as anti-inflammatory agents is around 0.7 and the Log*P* data around 4, which were similar to the theoretical probability of the common reference drugs diclofenac and indomethacin.

### 3.3. Nanoparticles Morphology

**Figure 3:**
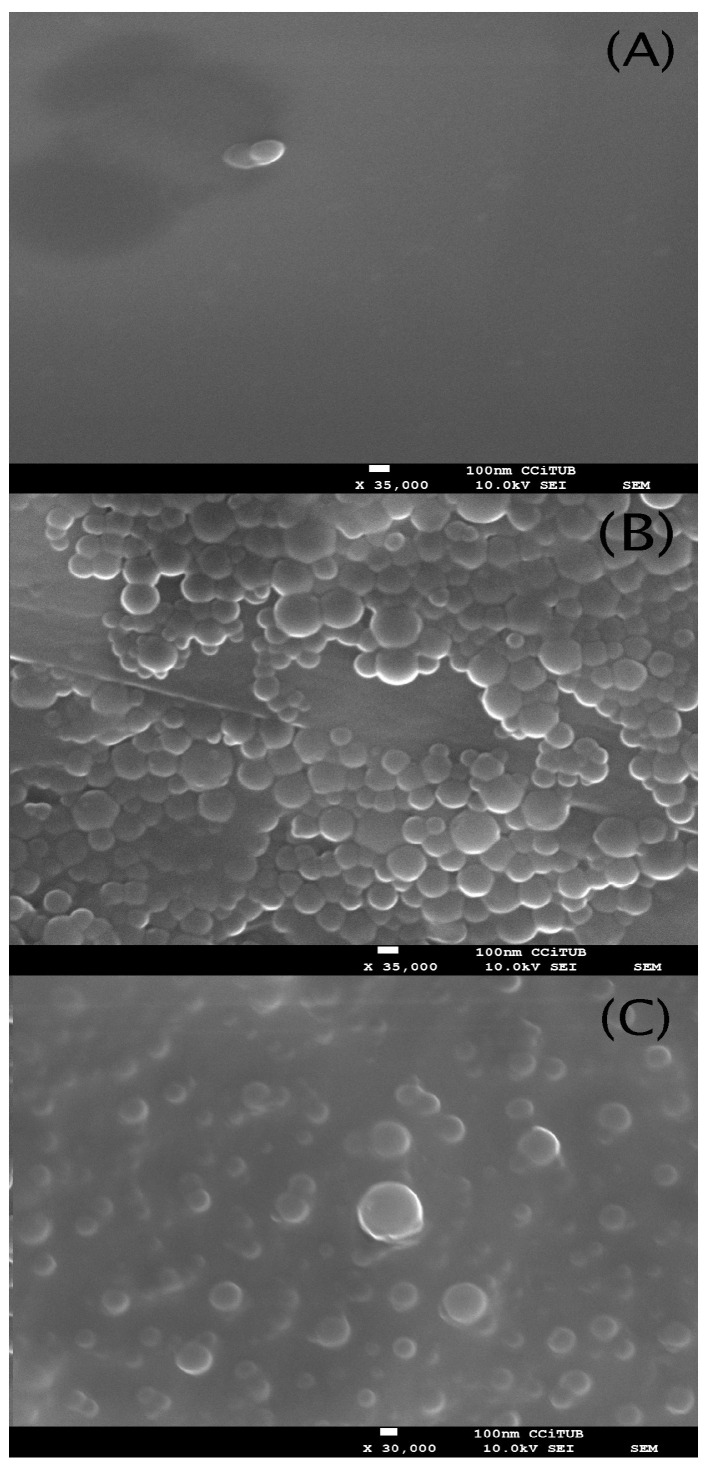
Scanning electron microscope (SEM) images obtained for: (**A**) nNP (**B**) NP I, and (**C**) NP II.

Scanning electron microscopy was used to evaluate the morphology of the nNP, NP I and NP II. In Figure 3, the 3D images of nNP, NP I and NP II morphology are displayed. They show spherical appearances with an average size of 144 ± 14.99 nm for nNP (155.42 nm maximum size and 134.21 nm minimum size), 118.18 ± 28.45 nm for NP I (194.68 nm maximum size and 60.03 nm minimum size) and 124.35 ± 34.11 nm for NP II (199.94 nm maximum size and 60.35 nm minimum size), respectively.

### 3.4. Nanoparticle Stability

**Figure 4:**
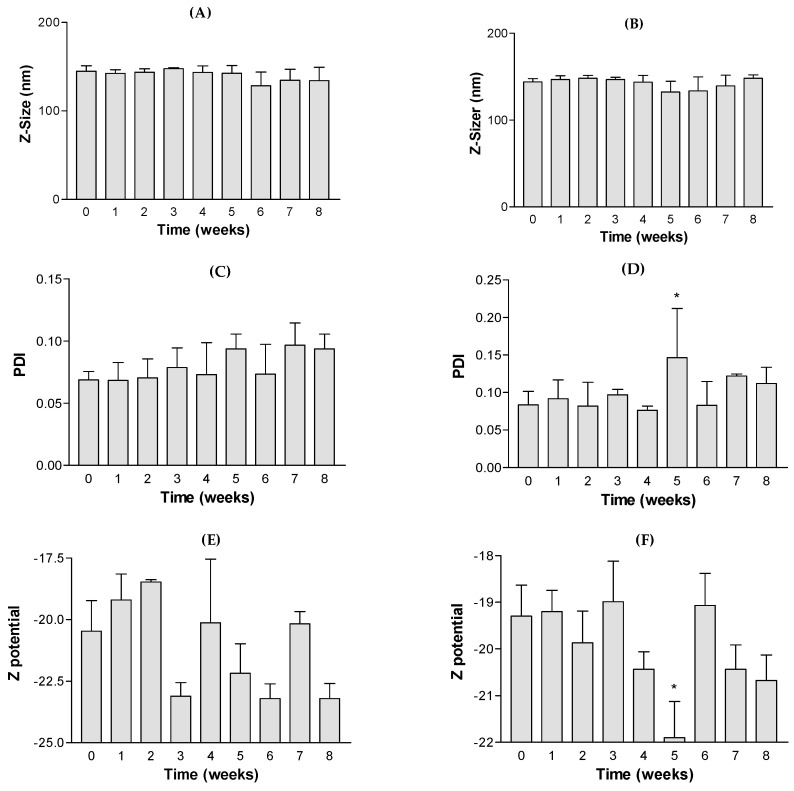
Stability analysis of flavanones NP formulations: (**A**) NP I Z-size data; (**B**) NP II Z-size data (**C**) NP I PDI = polydispersion index; (**D**) NP II PDI = polydispersion index; (**E**) NP I zeta potential data; (**F**) NP II ZP = zeta potential data (Mean ± standard deviation of 3 replicates). Significant differences with 0 week, *p* < 0.05 (*).

The NP stability was studied by observing any change in the size of the NPs during storage time at 4 °C for 8 weeks. To confirm this stability, the size (Z), the polydispersity index (PDI) and the zeta potential (ZP) parameters were examined every week. As can be seen in Figure 4A–F, no significant changes (*p* < 0.05) in Z, PDI and ZP values from NP I and NP II were detected until week 4. These data indicate that NPs were uniformly distributed in the system without flocculation or precipitation.

### 3.5. Osmolality and Transparency of Nanoparticles

**Figure 5:**
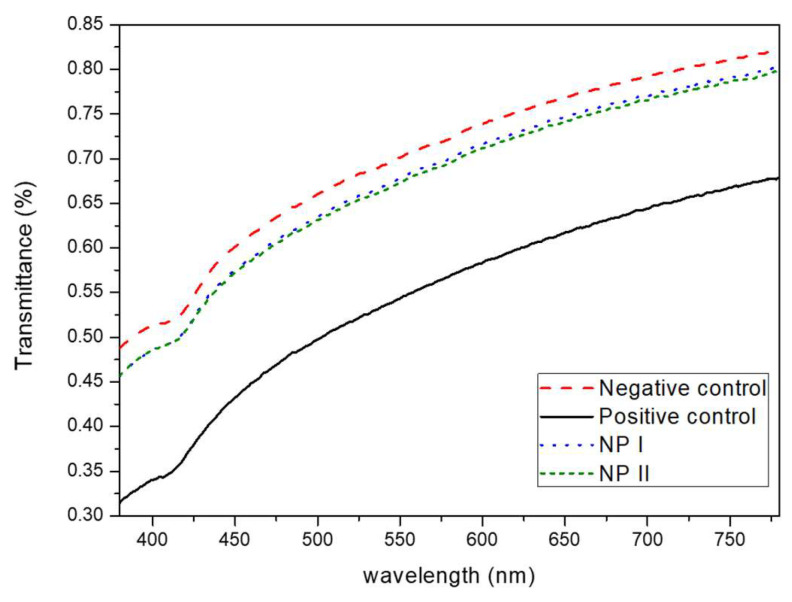
Graph of transmittance profile of corneas treated with physiological saline solution as negative control, 0.1 N NaOH as positive control, and NP I and NP II as the experimental formulations.

The osmolality for the samples studied was 283 mOsm/kg for nNP, 249 mOsm/kg per NP I and 301 mOsm/kg per NP II. Additionally, regarding the transparency of the NPs, NP I and NP II show transmittance values very similar to those of the negative control (physiological saline solution) unlike the positive control (0.1 N NaOH) that reduced corneal transparency. Furthermore, NP I and NP II have very similar transmittance profiles compared to each other indicating that both formulations do not affect corneal transparency (Figure 5). 

### 3.6. Ocular Irritation Study: HET-CAM

**Figure 6:**
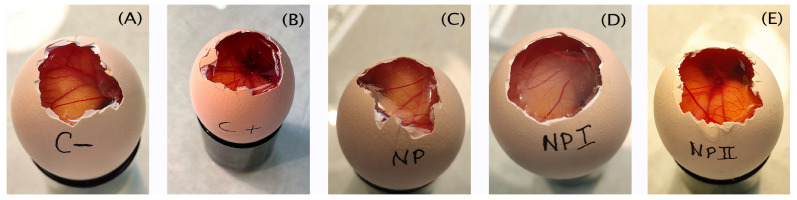
The effect of the studied formulations was evaluated using the HET-CAM method. (**A**) C−: negative control, saline solution, (**B**) C+: positive control, 0.1 N sodium hydroxide solution, (**C**) NP: nanoparticle with only excipients = nNP, (**D**) NP I: PLGA nanoparticle encapsulated flavanone I (**E**) NP II: PLGA nanoparticle encapsulated flavanone II.

The HET-CAM assay results presented in Figure 6 showed that after 5 min of treatment, the NP formulations did not cause any irritation on the chorioallantoic membrane of fertilized hen’s eggs, thus behaving as the negative control (C−, Figure 6A) and contrary to the positive control (C+) treated in which lysis was observed (Figure 6B). The irritation scores calculated for each sample were 6.74 ± 1.91 for C+ while they were 0.07 ± 0.00 for C−, nNP, NP I and NP II.

### 3.7. In Vitro Analysis: Release

**Figure 7:**
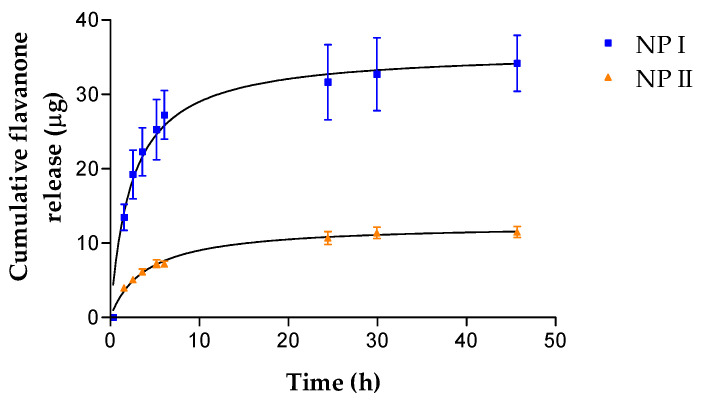
Cumulative amount released of flavanone I and II from NPs plotted against time.

The release profiles of flavanones I and II from the formulation PLGA NPs are presented in Figure 7. The best-fit kinetic model that describes the release profiles of flavanones I and II was obtained for a one-site binding hyperbola model with parameters shown in Table 3 (r^2^ = 0.9913 for I and r^2^ = 0.9957 for II). The formulation that released the largest amount of flavanone was NP I. Both parameters refer to velocity (*K_d_*) and release amount (*B_max_*) showed significant differences between NP I and NP II formulations.

### 3.8. Ex Vivo Analysis

The capacity of flavanones to diffuse through the cornea and sclera was evaluated by an ex vivo permeation study. Table 4 shows the amount of flavanones I and II retained at the end of the experiment (*Q_r_*, over 6 h). It was observed that the flavanones in the NP formulation were not capable of permeating these membranes (*Q_p_*).

Retention of flavanones I and II in the cornea and the sclera upon the application of NPs showed significant differences in the non-parametric Student’s *t*-test (*p* < 0.05). Moreover, local anti-inflammatory activity of the flavanones may be achieved.

### 3.9. Histology Evaluation

**Figure 8:**
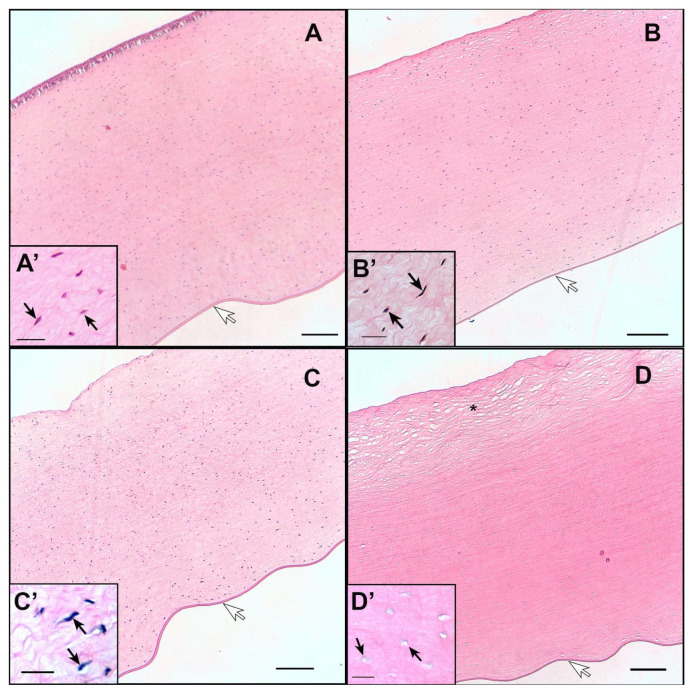
Representative histological sections of cornea: (**A**) treated with NP I; (**B**) treated with NP II; (**C**) negative control conditions treated with physiological saline solution; (**D**) positive control condition treated with 0.1 N NaOH. **A’**–**D’** = zoom sections of (**A**–**D**). Scale bar (**A**–**D**) = 200 μm and scale bar (**A’**–**D’**) = 25 μm. White arrow indicates the Descemet membrane; black arrows indicate cell nuclei and the asterisk indicates the more unstructured stroma area in the positive control.

**Figure 9:**
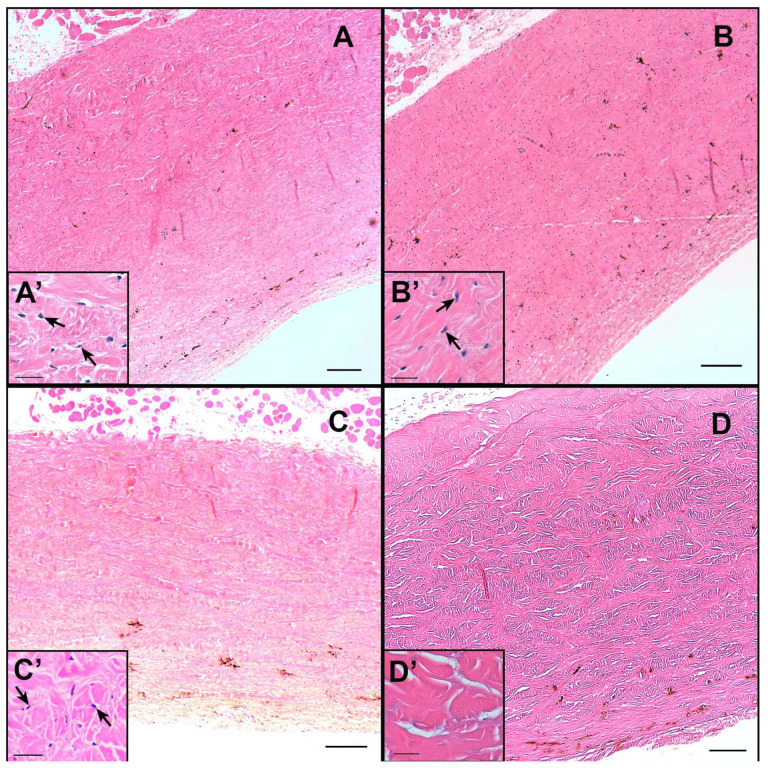
Representative histological sections of sclera: (**A**) treated with NP I; (**B**) treated with NP II; (**C**) negative control conditions treated with physiological saline solution; (**D**) positive control condition treated with 0.1 N NaOH. **A’**–**D’** = zoom sections of (**A**–**D**). Scale bar (**A**–**D**) = 200 μm and scale bar (**A’**–**D’**) = 25 μm. Black arrows indicate cell nuclei, which is present in almost all the figures except in the positive control.

Representative images of histomorphological analysis of assayed treatments on the cornea and sclera are shown in Figure 8 and Figure 9, respectively. The external epithelium (stratified squamous) is not observed in any preparation except with NP I treatment, in which it is partially preserved (Figure 8A). In practically all preparations, Descemet’s membrane is observed on the inner part of the cornea (Figure 8A–D, marked with the white arrow), but not the endothelial layer. Something similar occurred with the sclera samples, where it is not possible to see the choroid. 

The cornea samples were observed with fibrous cells, the keratocytes (Figure 8). In the positive control sample (Figure 8D’, marked with the black arrow) the lack of keratocytes (or that they are very deformed) was observed. While the samples (NP I, NP II and negative control—Figure 8A’–C’ marked with the black arrow) contained keratocytes and cell nuclei can be observed. The surface below the outer epithelium in the positive control image is the area that seems most unstructured compared to the other samples (Figure 8D’, marked with asterisk).

The sclera is a very fibrous tissue with cells, basically fibroblasts and some scattered pigment cells. No differences were observed between the negative control, NP I and NP II samples. Like the cornea samples, in the negative control, NP I and NP II samples (Figure 9A’–C’ marked with the black arrow) the cell nuclei are visible but in the positive control image the nuclei cannot be observed (Figure 9D’). The stroma in the positive control is unstructured to a certain degree, with spaces between the collagen fibers. This suggests that the positive control sample has undergone a more aggressive treatment than the rest.

### 3.10. Cytotoxicity Assays: Cell Culture and Cell Viability Assays

**Figure 10:**
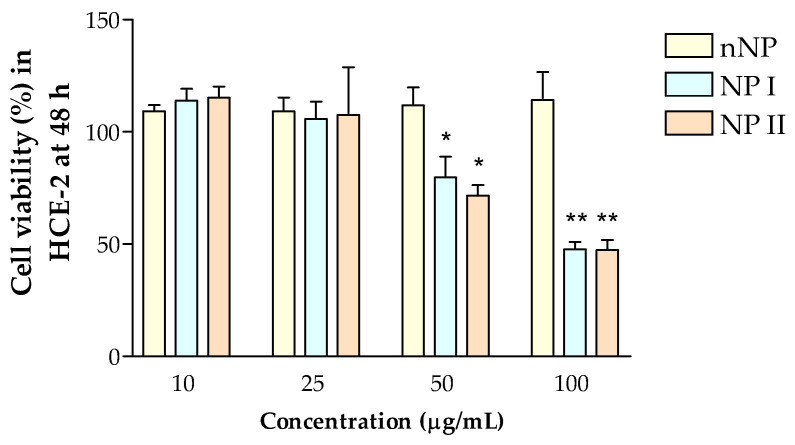
Cell viability of HCE-2 cells treated with the different formulations (nNP, NP I, NP II) at 48 h. Cell viability was assessed by the MTT assay and expressed as percentage of the viability of untreated control cells, assigned as 100%. Data are expressed as mean ± SD from three independent experiments. * *p* < 0.05 and ** *p* < 0.001.

To evaluate the cytotoxicity of NP I and NP II, HCE-2 cells were exposed to a concentration range of NPs (10 µg/mL–100 μg/mL) and incubated for 48 h. The NP stock solutions were diluted to obtain the indicated concentrations in terms of flavanone content. Cell survivals were assessed by 3-(4,5-dimethylthiazol-2-yl)-2,5-diphenyltetrazolium bromide (MTT) assay. (Figure 10). Results showed that the empty nNPs did not affect cell viability at any concentration evaluated indicating that the PLGA formulation was safe. On the other hand, NP I and NP II presented a significant cytotoxic effect on HCE-2 cells at concentrations higher than 50 μg/mL. Nevertheless, no toxicity was observed at concentrations up to 25 μg/mL. At these lower concentrations, both NP I and NP II yielded MTT values like those of untreated control cells.

### 3.11. In Vitro Anti-Inflammatory Analysis Determination of Pro-inflammatory Cytokines

**Figure 11:**
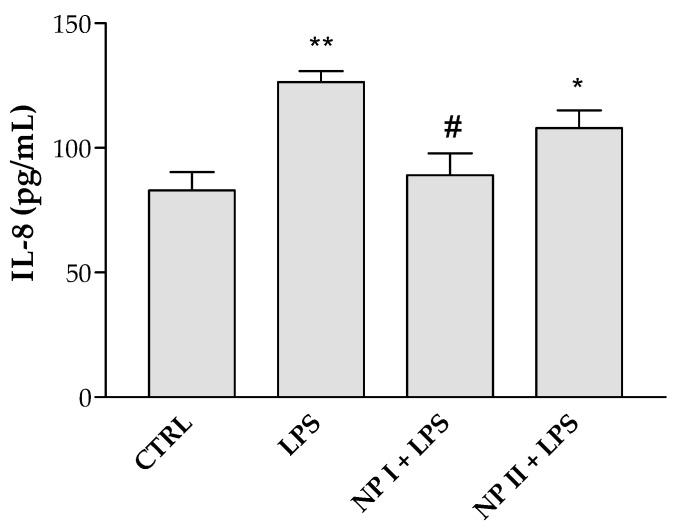
Effect of the NPs on the expression of the pro-inflammatory cytokine IL-8 in HCE-2 cells challenged with LPS. HCE-2 cells were pretreated with the different NPs at a final concentration of 25 µg/mL for 24 h prior to the addition of LPS (10 µg/mL). Cells which had not undergone LPS treatment were processed as a control (CTRL). IL-8 levels were quantified in the culture supernatant by ELISA following 24 h incubations with LPS. Data are presented as mean ± SD. Statistical significance: * *p* < 0.05 and ** *p* < 0.001 versus untreated control cells, # *p* < 0.001 versus LPS-treated cells.

HCE-2 cells treated with LPS were used as an in vitro model to evaluate the anti-inflammatory efficacy of NP I and NP II by measuring secreted levels of the pro-inflammatory cytokine IL-8. For both NPs, the 25 µg/mL dose was chosen for this study since it was not cytotoxic for HCE-2 cells (see Figure 10 or Section 3.10). As shown in Figure 11, LPS-treated cells secrete significantly higher levels of the pro-inflammatory cytokine IL-8 than untreated control cells (CTRL). Treatment with NP I significantly reduced IL-8 expression in LPS-treated cells, yielding IL values similar to those of untreated control cells. NP II was also able to diminish IL-8 production, although differences with respect to LPS-treated cells were not statistically significant.

## 4. Discussion

The interest in demonstrating the anti-inflammatory properties of flavanones demands the use of precise, reproducible, and sensitive analytical methods to quantify the new compounds that have not yet been validated. In this respect, the analytical method to quantify flavanones I and II was validated using high performance liquid chromatography (HPLC). The method developed resulted in being linear, with a precision, accuracy and instrumental repeatability within the previously established limits (RE% and RSD% < 15%). LOD for flavanones I and II were of the orders of 5.9 µg/mL and 1.8 µg/mL and LOQ of 17.8 µg/mL and 5.6 µg/mL, respectively. The computer web programs Molinspiration^®^ and PASS Online used in this work allowed the estimating of the probable profile of the anti-inflammatory activity of flavanones I and II compared with commercial drugs used for this purpose. The probability that flavanone could be active (*Pa*) up to 0.7, similar to the common drug references, suggests that they could be good candidates as anti-inflammatory agents (Table 2). Inflammation modulation has proven to be a useful therapeutic strategy for ocular surface diseases [35]. However, the hydrophobicity of flavanones limits their use in the treatment of inflammatory ocular diseases. Therapies based on the use of nanostructures have come to prominence in recent years [36]. Our work attempted to overcome this restriction by the incorporation of new compounds into nanoparticles. In this study, flavanones I and II were shown to be successfully entrapped in the NPs. In order to compare the flavanones, the same preparation method was used for both and thus in this way it was possible to reduce the variability due to the formulation. Flavanones PLGA NP formulations (NP I and NP II) produced by the displacement technique provided small particles with good homogeneity and high negative values of zeta potential indicating the stability of these systems. Moreover, the physical stability of the formulations was evaluated by observing possible changes in the physical appearance. In addition, the NP sizes were measured with Zetasizer equipment to verify if potential changes occurred during the storage at 4 ± 1 °C for 8 weeks. PDI values < 0.4 indicate a homogenous population [7]. The data obtained without significant size variations until week 4 indicated that the NP formulations examined were physically stable for one month. Lyophilization is recommended in subsequent studies to improve the long-term stability of PLGA NPs [37]. These data are consistent with those reported in some of the literature in which the presence of Poloxamer 188 in the PLGA nanoparticles loaded with flurbiprofen significantly improved the stability of the nanoparticles. Furthermore, the formulation was not irritating or toxic after its topical application in rabbits’ eyes [38]. Related to this question, Edelhauser et al. reported that corneal transparency or opacity decreased when the ocular cells of the endothelium were damaged due to a disease [39]. The transparency obtained from NP I and NP II was of a similar value and did not show a decrease in the transparency as in the positive control, which allows us to demonstrate that they do not cause any damage. The osmolality values of NPs were within the acceptance range of 250–450 mOsm/kg for topical ophthalmic administration [32,40], hence, no ocular irritation would be expected. Additionally, the HET-CAM assay showed that NP I and NP II did not cause acute irritation, therefore they are considered suitable for ophthalmic use. The HET-CAM assay is an alternative method to evaluate the ocular effects that appear due to the acute exposure of the compounds on the ocular mucosa, by observing the reactions that occur to the chorioallantoic membrane (CAM) of embryonated chicken eggs [41,42,43]. According to the physicochemical characteristics studied, these systems could be suitable for ocular application. Nevertheless, other methods that enable us to reveal further toxicological information must be assessed.

Another question is the studies of the in vitro flavanone release behavior of the NPs. Our results confirmed that flavanones could be released from the formulation. Since the components of NP I and NP II are the same except for the encapsulated flavanone, the significant differences shown in *K_d_* and *B_max_* could be due to the molecular structure of flavanones I and II. In the case of the ex vivo permeation study of NPs, neither flavanone I nor II was able to penetrate through either the cornea or the sclera. They were only retained in them. This effect could probably be due to the strong ability of flavanones to interact with the cornea and sclera. The presence of unbalanced charges (+/−) on the surface of the molecules can influence their permeability through the cornea and sclera [5,44]. It is known that the cornea has a negative charge on the surface; thus, cationic nanoparticles can be attracted owing to electrostatic interactions achieving topical drug delivery to the eye [45,46,47,48]. The effective surface charge of the nanoparticles, measured as zeta potential, is important because it allows their conjugation and retention at the ocular surface [49]. In the same way, it is known that for an efficient permeability of the corneal tissue, the molecules should be neither too lipophilic nor too hydrophilic, with a log octanol–water partition coefficient (log*P*) in the range of 2–3 [50]. According to the data obtained by the in silico analysis (Table 2) the miLog*P* of flavanones I and II was around 4. This is how it allows the possible anti-inflammatory effect of the flavanones to be exerted locally, preventing its accumulation in the aqueous humor and the vitreous. Topical administration for the treatment of ophthalmic disorders is preferred. It is convenient because it permits self-administration and minimizes the side effects of the systemic absorption of molecules [51].

The fact that NP I and NP II were not able to diffuse through corneal or scleral tissues could limit their ability to reduce inflammatory processes in certain regions of the eye. However, future modifications of the formulation strategy [52] or some structural modifications of I and II molecules should allow a better capacity of the candidate drug to reach posterior eye segments and to treat a wider group of ocular diseases with inflammatory features.

With respect to the histology evaluation, the effect of the positive control (Figure 8D and Figure 9D) was clearly observed., The fibers were preserved, but not the cells, in contrast to the other treatments (with NP I and NP II), in which both fibers and cells were preserved. This allows us to corroborate that the NP I and NP II do not cause apparent damage to ocular structures (cornea and sclera). Moreover, the absence of endothelium lining in the cornea and the choroid (Figure 8) suggests some type of unintended aggression that has caused its detachment or that it has not been preserved. Nevertheless, this cannot be attributed to a consequence of the treatments to which the samples under the positive control and NP I, NP II were subjected, since this phenomenon was also observed in the negative control samples. Rather, it may be due to the handling or processing of the samples. However, whatever is the case, this fact did not affect the result itself, or its validity, and it is demonstrated that they are innocuous in the treatments. As shown in the negative control, the NP I and NP II. HCE-2 cytotoxicity study showed no toxicity with NP I nor NP II below 25 µg/mL, which is consistent with the result of the ocular irritation study (HET-CAM). In fact, viability assays in HCE-2 cells ruled out any cytotoxic effect associated to NP I or NP II at doses below 25 µg/mL.

Ocular inflammation may be propagated due to the action of specific cytokines secreted in the tear covering the entire ocular surface epithelium [53,54]. Some flavonoids have been shown to inhibit various mediators that are activated in certain ocular inflammatory conditions (NO, prostanoids, leukotrienes, cytokines, and adhesion molecules). Among those mentioned immediately above, IL-6 and IL-8 play an important role in ocular inflammation [55]. With regards to this, it was reported that the flavone called Wogonin significantly suppressed IL-6 and IL-8 production on the ARPE-19 cell line [50]. Also, it was indicated that the flavonol quercetin triggered significant downwards regulation of IL-6 and IP-10 (interferon gamma inducible protein 10 kDa) secretion on human conjunctival and corneal epithelial cell lines [56,57]. In vitro studies carried out in epithelial cells of the human cornea treated showed that extracts of *Euphrasia officinalis* L. exercise anti-inflammatory activity by reducing the expression of pro-inflammatory cytokines (TNF-α, IL-6 and IL-1β) [9]. Here, we used the human corneal epithelial cell line (HCE-2), which is commonly used in ocular studies because these cells have a characteristic morphology, and formed a stratified epithelium that provides barrier properties [58]. Our results indicated that only the NP I formulation was capable of inhibiting LPS-induced production of interleukin 8. However, NP II displays a faint anti-inflammatory activity under the conditions tested in this work using the LPS inflammation model.

In summary, the NP I formulation could be a potential candidate for the treatment of ocular inflammation. Future research is needed to explore the anti-inflammatory effect of NP I and NP II in an in vivo animal model, as well as to elucidate the mechanism of action and discard potential side effects. These in vivo studies will allow the evaluation of the therapeutic efficacy of flavanone NPs as ocular treatments.

## 5. Conclusions

Two PLGA nanoparticles formulation were prepared with the natural flavanones I and II extracted from *E. platycarpa*. The analytical method of the quantification of flavanones was validated. The prediction of theoretical flavanone anti-inflammatory ability compared to diclofenac and indomethacin was also investigated, but in future studies this information will require support from experimental data. The release of flavanones from the PLGA NPs and the capacity for diffusing through the pig’s cornea and sclera was also evaluated. The release of flavanones followed a one-site binding hyperbola model, showing flavanone I to have a higher release than II. The NP I resulted in a higher retention of the flavanone in the sclera. However, neither NP I nor NP II could permeate the cornea and sclera. Hence, a higher amount of flavanone is available in the eye for a local effect. Both formulations were well-tolerated since they did not show any significant damage to the cornea and sclera in the histological study. Furthermore, no irritation was observed when the formulation was evaluated with the HET-CAM assay. Finally, the anti-inflammatory activity was assessed on HCE-2 cells where the NP I formulations evidenced the reduction in the pro-inflammatory cytokine IL-8. To sum up, the PLGA NP I formulation showed satisfactory properties for ocular application. However, additional studies are required to formulate a pharmaceutical presentation. 

## Figures and Tables

**Table 1:** Linearity, LOD, LOQ, accuracy, precision, (calculated at three levels of concentration) and repeatability of the analytical method using HPLC for determination of flavanones.

Flavanone	Linearity		LOD	LOQ	Accuracy	Precision	R.I.S
	r^2^	*p*-Value	(µg/mL)		RE (%)	RSD (%)	RSD (%)
			Mean ± SD		100 (µg/mL)		100 (µg/mL)
					25 (µg/mL)		
					6.25 (µg/mL)		
I	0.996	0.468	5.881 ± 2.202	17.820 ± 6.674	−0.586	1.244	7.609
					2.312	12.510	
					−3.774	15.336	
II	0.999	0.932	1.833 ± 1.090	5.556 ± 3.304	0.110	0.567	1.999
					−0.003	3.007	
					−2.718	4.623	

r^2^ = coefficient of determination at 100–6.25 (µg/mL). LOD = limit of detection; LOQ = limit of quantification; RE = relative error; RSD = relative standard deviation; R.I.S = repeatability of instrumental system.

**Table 2:** Theoretical calculation of physicochemical characteristics of flavanones I, II, diclofenac and indomethacin.

Data	Flavanone		Diclofenac	Indomethacin
	I	II		
Anti-inflammatory (*Pa*)	0.78	0.75	0.79	0.71
miLog*P*	4.81	4.87	4.57	3.99
TPSA	66.76	76	49.33	68.54
natoms	25	27	19	25
MW	338.40	368.43	296.15	357.79
Volume	316.14	341.69	238.73	303.24

*Pa*: probability of being active. miLog*P*: logarithm of octanol–water partition coefficient obtained by Molinspiration. TPSA: topological polar surface area. natoms: number of atoms. MW: molecular weight.

**Table 3:** One-site binding hyperbola release model parameters (mean ± Std. error).

	NP I	NP II	*p*
*B_max_* (µg)	35.9 ± 1.39	12.56 ± 0.39	0.002
*K_d_* (h)	2.38 ± 0.36	3.92 ± 0.41	0.001
r^2^	0.9913	0.9957	

*K_d_* = time needed to reach 50% of the drug released; *B_max_* = the maximum amount susceptible to release.

**Table 4:** Estimated permeation and retention parameters of flavanones NP I and NP II. Values are reported as the mean ± SD (*n* = 5).

	NP I		NP II	
	Cornea	Sclera	Cornea	Sclera
*Q_r_* (µg/cm^2^)	4.26 ± 0.57	7.85 ± 1.14	3.52 ± 0.40	5.09 ± 0.63
*Q_p_* (µg)	Non-p	Non-p	Non-p	Non-p

*Q_r_* = amount of flavanone retained in the cornea and sclera membrane; *Q_p_* = amount of flavanone permeated through the cornea and sclera membrane; Non-p: non-permeation was observed.

## Data Availability

Data are contained within the article.
